# Personalized cancer screening: helping primary care rise to the challenge

**DOI:** 10.1186/s40985-018-0083-x

**Published:** 2018-02-21

**Authors:** Kevin Selby, Gillian Bartlett-Esquilant, Jacques Cornuz

**Affiliations:** 1 Department of Ambulatory Care and Community Medicine, University of Lausanne, Lausanne, Switzerland; 2 Kaiser Permanente Division of Research, 2000 Broadway, Oakland, CA 94612 USA; 3 Department of Family Medicine, McGill University, 5858 chemin de la Côte-des-Neiges, 3rd floor, Montreal, Quebec H3S 1Z1 Canada

**Keywords:** Cancer screening, Personalized medicine, Population health, Primary care

## Abstract

With their longitudinal patient relationships, primary care physicians and their care teams are uniquely situated to promote preventive medicine, including cancer screening. A confluence of forces is driving the demand for the personalization of cancer screening recommendations. Recommendations are increasingly based on individual patient preferences, medical history, genetic and environmental risk factors, and level of interaction with the healthcare system. Current examples include choices between colonoscopy, fecal testing, and emerging tests for colorectal cancer (CRC) screening; the use of genetic information and availability of home self-testing in cervical cancer screening; the integration of multiple risk factors and patient preferences to decide the intensity and length of breast cancer screening; and the issues of smoking cessation and competing priorities when deciding whether or not to pursue lung cancer screening. These changes will inevitably increase the burden on primary care of providing high-quality cancer screening to their patients. To address, primary care physicians need access to continuously updated evidence reviews including prioritization of strongly supported recommendations, training in shared decision-making and tools for preference diagnosis, and an electronic health record (EHR) and reimbursement model that allow for population health management and team-based care. Only by reinforcing cancer screening in primary care can we ensure that personalized cancer screening is accessible and evidence-based.

## Background

### Vignette

Mrs. J, an active 55-year-old smoker in good health, comes to your primary care office with a few days of cough and no fever or shortness of breath. Looking through her chart, you realize that it has been 3 years since you last saw her. She had followed up for fasting blood work (all normal), but not the recommended cervical cancer screening, fecal occult blood test, flu vaccine, and dedicated visit to discuss smoking cessation. After discussing her acute bronchitis, she asks whether or not she should really have a mammogram, having read that it can increase testing and treatment without any benefit. She also mentions a recent article she read about preventive mastectomies and wonders if genetic testing will help with deciding. Already 25 min behind, you do not know where to begin discussing prevention with this patient. What could make this situation easier?

### Background

Primary prevention and screening of primary care patients are seen as a core part of appropriate care in the ambulatory setting [1]. As trusted advisors, primary care physicians (PCPs) and their care team are in an excellent position to administer guideline-recommended, age- and sex-specific preventive care and health promotion. Beginning with the first dichotomous recommendations of the Canadian Preventive Services Task Force in 1979, cancer screening recommendations have traditionally given levels of evidence to clearly support simple messages for testing or not testing broad swaths of the population. These recommendations were founded on the belief that evidence-based medicine would guide all decision-making and that simpler recommendations could ease implementation in daily practice. Multiple forces, however, have caused prevention recommendations to become more nuanced, as shown in Table 1. This evolution is likely to continue, with increased pressure to use population-level outreach to reach patients not seen in clinic, refine risk estimates using calculated scores and genetic data, and use information that directly targets the person to actively engage in their health [2]. Personalized screening regimens should improve the efficiency, equity, and safety of cancer screening but will require intensive input from primary care.

**Table 1:** Examples of increasing complexity in cancer screening recommendations and elements needed to allow for implementation of these recommendations into routine primary care

Cancer screening	Examples of personalization	Elements needed for implementation in routine practice	Current implementation examples
Breast	- Integration of personal preferences into whether to initiate screening and at what age - In the future, likely to have further integration of family history, screening history, and personal preferences for screening intensity	- Patient decision aids that present risk of overdiagnosis with screening - Electronic health record (EHR) decision support to allow tracking and integration of multiple patient factors	- Australian patient decision aid shown to increase informed choices [14] - Women Informed to Screen Depending on Measures of risk (WISDOM) study [22]
Colorectal	- Use of tailored outreach and inreach to ensure that all patients have the best opportunity possible to complete screening - Integration of patient preferences to choose screening modality	- EHR that clearly identifies patients not up to date with screening - Team-based care to reach patients who do not have regular appointments - Patient decision aids that present choice between screening modalities	- Kaiser Permanente screening program [21] - Canton of Vaud colorectal cancer screening program that integrates patient preferences [15]
Lung	- Balancing prevention messages of smoking cessation and early cancer detection - Risk stratification based on age, tobacco history, and possibly genetic and imaging findings - Integration of patient preferences when balancing potential benefits with substantial risk of overtreatment	- Recommendations that prioritize tobacco cessation ahead of lung cancer screening - EHR with structured tobacco history allowing for identification of eligible patients - Patient decision aids and conversation aids that quantify risks and benefits of screening and stimulate discussion - Reimbursement of time spent discussing screening decisions	- EviPrev recommendations that assign priority to proven effective prevention activities [17] - The Veterans Health Administration created patient decision aids and modified their EHR to support lung cancer screening [19]
Cervical	- Individualized screening intervals based on risk human papillomavirus (HPV) status, vaccination and risk factors - Individualized outreach offering multiple screening modalities to lower barriers to screening	- EHR with searchable vaccine history and pathology results - Targeted use of mailed, home self-screening despite lower accuracy	- Pilot studies integrating in-clinic and home HPV testing into organized screening in Italy [24]

### Current challenges

First, an increasing number of screening recommendations encourage shared decision-making, given the growing recognition of the complex trade-offs involved in cancer screening. Strong public health messages have presented cancer screening as a panacea of early detection of aggressive cancers at treatable stages; the reality is far more complex, with frequent overdiagnosis and the treatment of “cancers” never destined to cause symptoms. Breast cancer screening recommendations, for example, now urge physicians to tailor screening based on patient preferences, especially for women outside of the 50 to 75 age range and those with dense breasts. Different women may approach the balance of marginally reduced breast cancer mortality and risks of being treated for pseudo-disease differently, making it essential that PCPs know how to “diagnose” their patients’ preferences [3]. It is challenging for organized, mass screening programs to integrate these messages in their mailed materials, and PCPs can offer a place for more nuanced discussions. Screening tests that are not incorporated into mass screening because of controversies regarding their efficacy and substantial harms, such as prostate cancer screening, are also ideally addressed in the setting of a longitudinal, trusting relationship [4].

Second, screening recommendations increasingly require detailed patient information, often in the form of clinical risk scores or genetic testing, to determine whether screening is indicated and at what intensity [5]. Lung cancer screening, for example, is only indicated for adults aged 55 to 80 years who have a 30 pack-year smoking history and currently smoke or have quit within the past 15 years [6]. Such a specific patient population precludes mass mailings without access to individual medical history. Similarly, cervical cancer screening recommendations increasingly integrate patients’ vaccination history and past human papillomavirus (HPV) history so as to maximize cancer detection while reducing the substantial number of false positives with annual cytology [7]. Genetic information combined with detailed clinical information for breast cancer screening is now enlarging the range of at-risk women while at the same time presenting the challenging topic of risk stratification which inherently implies that low or at population risk women might benefit from less screening than currently recommended.

Third, individual screening tests are only one part of an increasingly long list of available tests and recommendations that are indicated for multimorbid patients. One widely cited article calculated that PCPs in the USA would need 7.4 h a day just to complete all prevention activities for an average panel of patients, in addition to the 3.5 h a day to manage ten common chronic diseases, an obviously impossible task [8, 9]. Not all of the recommendations, however, offer the same certainty of benefit for individual patients; choices therefore need to be made, and we are not always giving the right messages in the first place. For example, overestimation of the benefits of lung cancer screening can actually detract from smoking cessation efforts, while substantial evidence supports interventions for smoking cessation ahead of screening in terms of cancer prevention [10].

Fourth, PCPs are increasingly expected to demonstrate their public health impact by improving cancer screening rates for their population of patients, particularly for strongly recommended tests like with colorectal cancer (CRC) screening [11]. There is evidence that PCPs can increase screening rates, even in the presence of organized screening programs [12].

### Reinforcing primary care

**Fig. 1:**
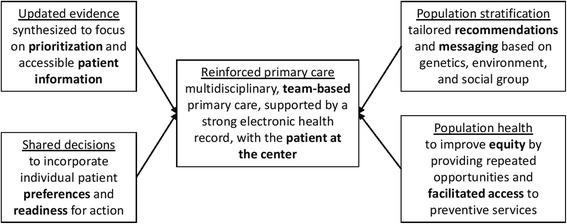
Elements needed to support primary care to make personalized cancer screening possible

Though our vignette might be viewed as too familiar to PCPs, it illustrates some features of the current situation in primary care. Limited resources prevent in-depth discussions, stratification of patient populations, and contact with patients between visits. While PCPs have always used their in-depth knowledge of their patients’ personal situation to personalize care, an increasing number of complex recommendations, rising expectations, and rapidly developing technology and innovations are increasing strain [13]. We need to reinforce primary care if we want all patients to have access to personalized cancer screening (Fig. 1). Numerous examples exist of how primary care can be augmented to make personalized cancer screening a reality (Table 1).

Most people who participate in cancer screening are unaware of the harms that need to be evaluated in perspective of potential benefits. Patient decision aids, as demonstrated in a recent randomized trial [14], can help patients understand overdiagnosis and prepare them for discussions with their PCPs. Concurrently, PCPs need time and training to engage their patients in difficult discussions rather than present screening for breast, prostate, and lung cancer as essential services that are to be completed by obedient patients. Several CRC screening programs, including the one we developed in a French-speaking canton of Switzerland, provide patients with materials to aid with the choice between fecal occult blood testing and colonoscopy; our local program even incorporates a reimbursed PCP visit to ensure that high-risk patients are identified and that average-risk patients have an opportunity to discuss their options [15]. A pilot program showed that training PCPs with this approach increased the proportion intending to use both targeted screening tests in their practice [16].

Prevention recommendations need to be regularly updated and differentiate which ones should come first and receive the strongest endorsement. These recommendations need to be nuanced with clear directions on how to incorporate family history and genomic testing to present well-defined risk profiles. Following similar developments in other countries, the EviPrev programme in Switzerland attempts to assign clear priority to prevention activities with evidence of substantial benefit using an interactive online table that is linked to information resources that can be given to patients [17].

Such work needs to be supported with computerized decision support tools and regularly updated lists showing which prevention tasks should be discussed first, to encourage PCPs to deliver difficult messages and to aid PCPs in understanding more complex information that usually accompanies genetic-risk stratification [18]. The implementation of lung cancer screening in the Veterans Health Administration provides an example of using an electronic health record (EHR) to navigate complex screening recommendations [19]. Clinical reminders were created to remind providers to enter tobacco history and current smoking status, to discuss the risk and benefits of screenings with patients identified using an electronic algorithm, and to aid with the provision of appropriate follow-up and repeat screening. Even after the implementation of these electronic tools, however, the collection of required information demanded substantial staff resources, and the extent of shared decision-making was unclear. The PERSPECTIVE group is developing public and health professional communication tools to accompany their expansion of the BOADICEA model for genetic risk-stratification for women and breast cancer screening [20].

Kaiser Permanente Northern California provides an example of how PCPs can work in cooperation with organized screening programs to increase the use of highly recommended screening tests, such as fecal occult blood testing and colonoscopy for CRC screening [21]. The centralized CRC screening program uses EHR data to automatically send outreach materials to eligible patients, including a PCP-signed pre-letter, a fecal immunochemical test kit, robo-call reminder, and a reminder postcard. If patients complete the fecal test, their EHR is updated in real-time. When patients present for care at any Kaiser location, all members of the healthcare team have access to the population-tracking software and can encourage participation. Lists are also created of patients not up to date with screening to provide personalized inreach by sending secure electronic messages and making phone calls.

### Future directions

The so-called *precision medicine revolution* has the potential to improve the early detection of cancer while simultaneously increasing precision, furthering current trends towards personalized screening regimens. Although these challenges were noted in the current screening context, to ensure the appropriate information, education and clinical information is provided for the primary care context, PCPs will need to play a critical role in these changes to be able to successfully integrate genetic testing and patient preferences [13]. The WISDOM trial may provide a glimpse of the not-so-distant future [22]. Women in the intervention arm will use a combination of clinical risk factors, breast density, and a polygenic risk score representing the cumulative effect of multiple single nucleotide polymorphisms (SNPs) and sequencing for moderate- and high-penetrance germline mutations to decide whether they choose more or less intensive breast cancer screening based on frequency of mammography and use of adjunctive magnetic resonance imaging. Importantly, women will always be given the option of pursuing more intensive screening and informed of the uncertainty surrounding personalized screening. The investigators hope to enroll 100,000 women and expect that a significant proportion of women will choose low-intensity screening, possibly reducing the impacts of overdiagnosis, while still identifying high-risk women. For precision cancer screening such as this becomes widespread, PCPs will need extensive training and support [23].

## Conclusions

The push for personalized cancer screening recommendations has the potential to increase workload and complexity for PCPs. An increasing number of recommendations recognize the complex trade-offs in screening and call for the incorporation of patient preferences. Detailed patient and family history-taking is required to identify patients who are most likely to benefit and for whom screening is indicated. Simultaneously, the large number of potential prevention activities makes prioritization key, and PCPs are expected to increase screening rates for their entire patient population.

The PCP in the vignette is struggling to provide excellent preventive care to a patient who rarely presents for appointments and simultaneously has multiple indicated tests. Examples exist, however, of how leading organizations are making personalized cancer screening routine, even for patients who do not see their PCP regularly. Patient decision aids can provide guidance; EHR can provide real-time decision support; integrated recommendations can help prioritize what to address first; and team-based care can help contact patients so that they can get screening tests when they are ready. These interventions can be used to not only address current challenges, but also prepare PCPs to incorporate the coming precision medicine era advances into their daily practice.
